# Prevalence, clinical presentation, and etiology of myelopathies in 224 juvenile dogs

**DOI:** 10.1111/jvim.17045

**Published:** 2024-03-14

**Authors:** Ed J. Pilkington, Steven De Decker, Eleftheria Skovola, Ana Cloquell Miro, Rodrigo Gutierrez Quintana, Kiterie M. E. Faller, Albert Aguilera Padros, Rita Goncalves

**Affiliations:** ^1^ Small Animal Teaching Hospital, Institute of Veterinary Science University of Liverpool Liverpool United Kingdom; ^2^ Department of Clinical Science and Services, Royal Veterinary College University of London Hatfield United Kingdom; ^3^ Small Animal Hospital, School of Biodiversity, One Health and Veterinary Medicine University of Glasgow Glasgow United Kingdom; ^4^ The Royal (Dick) School of Veterinary Studies The University of Edinburgh Midlothian United Kingdom

**Keywords:** diverticulum, malformation, screw‐tail, spinal

## Abstract

**Background:**

Intervertebral disc herniation is widely recognized as the most common cause of myelopathy in dogs older than 2 years; however, the prevalence of various causes of myelopathy in younger dogs has not been reported.

**Hypothesis/Objectives:**

To describe the prevalence, clinical presentation, and etiology of myelopathy in dogs aged 18 months or less. Secondarily, to investigate which clinical features were associated with each of the most common etiologies.

**Animals:**

Two hundred twenty‐four dogs aged 18 months or less with myelopathy were included in the study.

**Methods:**

Retrospective review of clinical records from 4 referral institutions. Multivariable logistic regression analyses assessed which clinical features were associated with each diagnosis.

**Results:**

French bulldogs (n = 51, 22.8%), pugs (n = 18, 8.0%), crossbreeds (n = 12, 5.4%), and English bulldogs (n = 11, 4.9%) were the most frequently affected breeds. Overall, 31 diagnoses were reached. The 5 most frequent diagnoses were vertebral malformation (VM; n = 42, 18.8%), spinal arachnoid diverticulum (SAD; n = 28, 12.5%), traumatic fracture of the vertebral column (n = 22, 9.8%), atlantoaxial instability (n = 18, 8.0%), and osseous‐associated cervical spondylomyelopathy (n = 17, 7.6%). Intervertebral disc extrusion (IVDE) accounted for 4.5% of cases (n = 10). A final diagnosis of VM was associated with younger, screw‐tailed, and pug breeds with chronic signs of T3‐L3 myelopathy. SAD was associated with screw‐tailed and pug breeds with nonpainful clinical signs. Intervertebral disc extrusion was associated with older, screw‐tailed, and pug breeds with shorter duration of clinical signs.

**Conclusions and Clinical Importance:**

Prioritization of differential diagnoses for dogs presenting with signs of myelopathy when aged 18 months or less should differ to those for older dogs, with IVDE not the most common cause in the former.

**Abbreviations**
aPTT: activated partial thromboplastin time
BMBT: buccal mucosal bleeding time
CM: constrictive myelopathy
IVDE: intervertebral disc extrusion
MMUO: meningomyelitis of unknown origin
OA‐CSM: osseous‐associated cervical spondylomyelopathy
OSPT: 1‐stage prothrombin time
PCR: polymerase chain reaction
SAD: spinal arachnoid diverticulum
T2W: T2‐weighted
VM: vertebral malformation

## 1 INTRODUCTION

Diseases of the vertebral column causing myelopathy in dogs are numerous.^1^ Within this broad group of conditions causing spinal cord injury, intervertebral disc extrusion (IVDE) is the most common underlying cause,^2^, ^3^ comprising 29.8% of cases referred with suspected spinal cord disease.^4^ Intervertebral disc extrusion typically occurs in dogs older than 2 years of age.^5^, ^6^

To date, the literature detailing the intersection specifically between juvenile dogs and myelopathies is limited. Publications have either been restricted to specific conditions that are well‐recognized in young dogs, such as atlantoaxial instability, fibrocartilaginous embolic myelopathy, vertebral malformations, cervical spondylomyelopathy, spinal arachnoid diverticula (SAD), and spina bifida,^7^, ^8^, ^9^, ^10^, ^11^, ^12^ or to isolated case reports including nephroblastoma, microchip implantation, and an inflammatory myofibroblastic tumor.^13^, ^14^, ^15^ Other studies have evaluated specific conditions across all age groups, with various findings relating to age, such as an increased likelihood of meningomyelitis of unknown origin in younger dogs,^16^ that the age of onset of clinical signs in dogs with spinal arachnoid diverticulum undergoing surgical management (median 18 months) was less than those undergoing medical management (45 months),^17^ and that steroid‐responsive meningitis‐arteritis (with or without signs of myelopathy) was more likely in younger animals (median age 11 months) when compared with meningomyelitis of unknown origin (52 months) or infectious disease (76 months).^18^ However, despite extensive literature on specific conditions that can affect younger dogs, the prevalence and underlying causes of juvenile myelopathies in dogs referred for signs of myelopathy remains unknown. Knowledge of likely causes allows clinicians to prioritize differential diagnoses, thus assisting with the formulation of an appropriate diagnostic plan. In cases where further investigations are not possible, this knowledge also enables clinicians to better inform clients regarding suitable empiric symptomatic treatment and possible prognosis.

Our study aimed to describe the prevalence, presenting signs, and etiology of myelopathy in dogs aged 18 months or less. Furthermore, we aimed to identify if any clinical features were associated with the most common diagnoses. We hypothesized that intervertebral disc extrusion would be uncommon in juvenile dogs.

## 2 MATERIALS AND METHODS

### 2.1 Study criteria

Retrospective review of medical records was undertaken at the small animal hospitals of the University of Liverpool, the Royal Veterinary College, the University of Glasgow, and the University of Edinburgh. Databases were searched to identify dogs that presented to the respective neurology services in the period between September 1, 2017 and September 1, 2022 with signs of myelopathy when aged 18 months or less. Signs of myelopathy comprised neurologic deficits that localized to 1 or more spinal cord segments, as determined by a board‐certified neurologist. In addition, a final diagnosis must have been reached after further investigations in order to be included in the study. Dogs were excluded if they presented exclusively with hyperesthesia on palpation of the vertebral column (and therefore without neurologic deficits) or if a final diagnosis could not be reached. In all cases, imaging of the vertebral column was performed (radiography, computed tomography, magnetic resonance imaging, or a combination of these modalities). In each case, the final diagnosis was determined through a combination of anamnestic, clinical, and diagnostic examination findings, including review of specialist‐written imaging reports. As deemed clinically necessary, additional diagnostic tests were undertaken to achieve the diagnosis, including cerebrospinal fluid analysis, hematology, serum biochemistry, coagulation profiles (including 1‐stage prothrombin time [OSPT], activated partial thromboplastin time [aPTT], and buccal mucosal bleeding time [BMBT]), urinalysis, infectious disease testing (serology, polymerase chain reaction, and antimicrobial culture methods), cytologic and histologic assessment of ultrasound‐guided and surgical biopsies, electrophysiologic assessment, and postmortem investigation. Vertebral malformation was considered responsible for the myelopathy when, in addition to the malformation substantially altering the course and dimensions of the vertebral canal, there were additional imaging features to support chronic disease (such as adjacent focal T2‐weighted [T2W] intramedullary hyperintensity), and in the absence of other identified causes of myelopathy. Morphologies of vertebral malformation within the eponymous final diagnosis group included both those that affected the vertebral body, such as hemivertebrae and block vertebrae, and the lamina, such as osseous stenosis secondary to articular facet hyperplasia. A final diagnosis of osseous‐associated cervical spondylomyelopathy (OA‐CSM) was assigned to large or giant breed dogs with myelopathies secondary to osseous stenosis caused by dorsal lamina or articular facet hyperplasia in the cervical vertebral column. Spina bifida, although also a vertebral malformation, was considered a distinct diagnosis because of its dorsal midline location, arising from aberrant neural tube closure. Based on the dogs identified in our study, a diagnosis of cranial cervical traumatic contusion was made in cases where signs resolved without treatment after suspected cervical hyperextension injury, and magnetic resonance imaging had identified a noncontrast enhancing T2W hyperintense intramedullary lesion at the level of the atlantooccipital joint, without evidence otherwise to suggest disc disease or atlantoaxial instability. Cases where signs of myelopathy were considered to feasibly result from more than 1 distinct condition were diagnosed with multiple diseases.

### 2.2 Data collection

Data collected from the medical records included age at presentation (months), sex, neuter status, breed, bodyweight (kilograms), onset, progression and duration of clinical signs (days), pyrexia (greater than 39.2°C), presence of systemic clinical signs, spinal cord segments affected (C1‐C5, C6‐T2, T3‐L3, L4‐S3, multifocal), presence of spinal hyperesthesia, ambulatory status, presence of ataxia, presence of postural reaction deficits, presence of either urinary or fecal incontinence or both, presence of lateralizing clinical signs, nociception, presence of spinal shock, imaging modality used, location of the lesion(s), and final diagnosis. Screw‐tailed breeds included the French Bulldog, English Bulldog, and Boston Terrier. Onset was categorized as acute if signs were clinically evident within 72 hours, or as chronic if greater than 72 hours. Progression was categorized as either static, deteriorating, improving, or waxing and waning; the category was determined at the point of presentation through a combination of the owner's report and clinical records from the referring veterinarian. Systemic clinical signs included hyporexia, lethargy, and signs of gastrointestinal disease. Spinal shock was deemed present in cases where the segmental spinal reflexes caudal to the lesion were transiently reduced, despite the spinal cord segments responsible for the reflex arc remaining unaffected by the identified lesion. Signs were deemed to be lateralized when there was a distinct difference in the neurologic deficits on the left and right sides of the body.

### 2.3 Statistical analysis

Statistical analysis was undertaken by a standard statistical software package (IBM SPSS Statistics for Mac, version 29, IBM Corp, Armonk, New York). For etiologies with 10 or more cases, univariable binary logistic regression was performed to identify if the following clinical features were associated with each final diagnosis: age, bodyweight, whether the dog was a screw‐tailed or pug breed, onset of clinical signs, duration of clinical signs before presentation, spinal cord segments affected, presence of spinal hyperesthesia, lateralization of clinical signs, ambulatory status, and presence of incontinence (urinary or fecal or both). For the regression analysis of each final diagnosis, the remaining study population without that diagnosis acted as the control group. Any independent variable demonstrating liberal association on preliminary univariable analysis (*P* < .25) was considered for inclusion in a multivariable model. Before performing the multivariable regression analysis, Pearson correlation coefficients were calculated to exclude any related variables (*r* > 0.8). The multivariable model was constructed with a manual backwards stepwise removal approach; variables with *P*‐value <.05 were retained as statistically significant.

## 3 RESULTS

### 3.1 Signalment

Two hundred twenty‐four dogs were included in the study with a median age at presentation of 8 months (range, 3 weeks‐18 months; IQR, 5 months). Eighty‐nine (39.7%) were female, of which 13 were neutered, and 135 (60.3%) were male, of which 29 were neutered. Median bodyweight was 10.9 kg (range, 0.6‐76.0 kg; IQR, 11.9 kg). In total, 62 breeds were represented; the most frequent breed was the French Bulldog (n = 51; 22.8%) followed by Pug (n = 18; 8.0%), crossbreeds (n = 12; 5.4%), English Bulldog (n = 11; 4.9%), Chihuahua (n = 7; 3.1%), Great Dane (n = 7; 3.1%), Staffordshire Bull Terrier (n = 7; 3.1%), Labrador Retriever (n = 6; 2.7%), and Dogue de Bordeaux (n = 5; 2.2%). Across all dogs, 83 (37.1%) were classed as a screw‐tailed or pug breed.

### 3.2 Presenting clinical signs

Onset of clinical signs was considered acute (developing in less than 72 hours) in 101 dogs (45.1%) or chronic (>72 hours) in 123 dogs (54.9%). Median duration of clinical signs before presentation was 15 days (range, 0‐455 days; IQR, 51 days). Signs were most frequently considered to be deteriorating (n = 139; 62.1%), followed by static (n = 64; 28.6%), improving (n = 10; 4.5%), and waxing and waning (n = 9; 4.0%). Systemic signs were reported in 29 dogs (12.9%), and pyrexia was noted in 8 dogs (3.6%).

Signs of myelopathy most frequently localized to the T3‐L3 spinal cord segment (n = 103; 46.0%), followed by C1‐C5 (n = 59; 26.3%), C6‐T2 (n = 23; 10.3%), multifocal (n = 21; 9.4%), and L4‐S3 (n = 18; 8.0%). Conscious pain perception was absent in 6 dogs (2.7%), of which all were paraplegic with 4 localizing to the T3‐L3 spinal cord segment and 2 with a multifocal neuroanatomic localization. Gait assessment was possible in 222 dogs, with 2 not assessed because of being immobilized after trauma. Of those assessed, 35 dogs (15.8%) displayed no paresis, 128 dogs (57.7%) were ambulatory paraparetic, 49 dogs (22.1%) were nonambulatory paraparetic, and 10 dogs were paraplegic (4.5%). Within the group of ambulatory dogs, the majority displayed proprioceptive ataxia (n = 136; 83.4%). Excluding the dogs without voluntary movement, the majority displayed postural reaction deficits (n = 183; 86.3%). In 45 dogs (20.1%), neurologic deficits were clearly lateralized. Spinal hyperesthesia was recorded in 36.2% of dogs (80/221). Spinal shock was noted in 8 dogs (3.6%), of which 5 were paraplegic, 2 were nonambulatory paraparetic, and 1 was ambulatory paraparetic. Incontinence was recorded in 22 dogs (9.8%), of which 9 were urinary incontinent, 2 were fecally incontinent, and 11 were both. Information regarding the clinical presentation of dogs with each of the most common final diagnoses is available in Table 1.

**TABLE 1:** Prevalence of presenting clinical signs for each of the most common final diagnoses of dogs with signs of myelopathy when aged 18 months or less.

	VM		SAD		Fracture		AAI	
	n (/42)		n (/28)		n (/22)		n (/18)	
Age (months)		42		28		22		18
Median (IQR)	6.0 (3.0)		7.5 (4.8)		8.5 (5.3)		7.2 (5.8)	
Bodyweight (kg)		39		28		22		18
Median (IQR)	9.7 (5.8)		8.9 (6.8)		12.1 (12.8)		3.8 (4.9)	
Breed		42		28		22		18
Screw‐tailed and pug breed	31		18		1		1	
Nonscrew‐tailed or pug breed	11		10		21		17	
Onset		42		28		22		18
Acute	10		4		20		10	
Chronic	32		24		2		8	
Duration of clinical signs (days)		41		28		22		18
Median (IQR)	21.0 (49.5)		60.0 (89.8)		1.0 (1.0)		21.5 (73.5)	
Progression		41		28		22		18
Static	7		11		15		4	
Deteriorating	31		16		6		11	
Improving	1		1		1		1	
Waxing‐waning	2		0		0		2	
Spinal cord segment		42		28		22		18
C1‐C5	4		13		4		16	
C6‐T2	0		0		1		0	
T3‐L3	35		14		13		1	
L4‐S3	2		0		4		0	
Multifocal	1		1		0		1	
Ambulatory status		42		28		20		18
Ambulatory	40		27		7		13	
Nonambulatory	2		1		13		5	
Spinal hyperesthesia		41		28		22		17
Painful	7		2		17		8	
Nonpainful	34		26		5		9	
Lateralization of clinical signs		42		28		21		18
Symmetrical	36		26		19		11	
Lateralized	6		2		2		7	
Incontinence		42		28		22		18
Continent	38		24		20		18	
Incontinent	4		4		2		0	

	OA‐CSM		MMUO		IVDE		Discospondylitis	
	n (/17)		n (/11)		n (/10)		n (/10)	
Age (months)		17		11		10		10
Median (IQR)	9.0 (6.5)		9.0 (3.7)		17.5 (6.0)		8.5 (3.8)	
Bodyweight (kg)		17		11		10		10
Median (IQR)	31.0 (27.4)		10 (11.7)		13 (2.8)		11.9 (14.0)	
Breed		17		11		10		10
Screw‐tailed and pug breed	0		6		8		5	
Nonscrew‐tailed or pug breed	17		5		2		5	
Onset		17		11		10		10
Acute	5		9		10		4	
Chronic	12		2		0		6	
Duration of clinical signs (days)		15		11		10		10
Median (IQR)	28.0 (53.0)		2.0 (13.0)		1.0 (1.3)		21.0 (48.0)	
Progression		16		11		10		10
Static	1		2		2		1	
Deteriorating	13		9		7		8	
Improving	1		0		0		0	
Waxing‐waning	1		0		1		1	
Spinal cord segment		17		11		10		10
C1‐C5	7		1		2		0	
C6‐T2	6		0		0		0	
T3‐L3	2		7		8		5	
L4‐S3	0		0		0		2	
Multifocal	2		3		0		3	
Ambulatory status		17		11		10		10
Ambulatory	15		8		5		7	
Nonambulatory	2		3		5		3	
Spinal hyperesthesia		17		11		10		10
Painful	3		3		7		8	
Nonpainful	14		8		3		2	
Lateralization of clinical signs		17		11		10		10
Symmetrical	14		7		9		10	
Lateralized	3		4		1		0	
Incontinence		17		11		10		10
Continent	16		10		10		10	
Incontinent	1		1		0		0	

Abbreviations: AAI, atlantoaxial instability; IQR, interquartile range; IVDE, intervertebral disc extrusion; MMUO, meningomyelitis of unknown origin; OA‐CSM, osseous‐associated cervical spondylomyelopathy; SAD, spinal arachnoid diverticulum; VM, vertebral malformation.

### 3.3 Final diagnoses

In total, 31 etiologies of myelopathy in dogs aged 18 months or less were identified. The most frequent final diagnosis was vertebral malformation (including hemivertebrae, butterfly, wedge, and block vertebrae; n = 42; 18.8%) followed by spinal arachnoid diverticulum (n = 28; 12.5%), traumatic fracture of the vertebral column (n = 22; 9.8%), atlantoaxial instability (AAI; n = 18; 8.0%), osseous‐associated spondylomyelopathy (n = 17; 7.6%), meningomyelitis of unknown origin (MMUO; n = 11; 4.9%), discospondylitis (n = 10; 4.5%), and intervertebral disc extrusion (n = 10; 4.5%). A complete list of final diagnoses is presented in Table 2. Within the group of 22 dogs diagnosed with a fracture of the vertebral column, in 20 (90.9%) cases, the owners witnessed or reported a traumatic event, with a further traumatic event suspected in 1 dog and 1 fracture occurring during exercise. Within the group of 6 dogs with absent conscious pain perception, 2 were diagnosed with a traumatic fracture of the vertebral column, 2 with intervertebral disc extrusions, 1 with hemophilia A, and 1 with meningomyelitis of unknown origin. Within the group of 5 dogs that had more than 1 underlying cause of myelopathy, 1 dog was diagnosed with vertebral malformation and myelodysplasia, 1 dog with vertebral malformation and spina bifida, 1 dog with atlantoaxial dorsal band with suspected secondary cervical syringohydromyelia, thoracic vertebral canal stenosis causing spinal cord compression, and meningomyelitis of unknown origin, 1 dog with thoracic vertebral malformation and discospondylitis, and 1 dog with syringohydromyelia of unknown cause, spina bifida, and thoracic vertebral malformation.

**TABLE 2:** Prevalence of final diagnoses for 224 dogs presenting with signs of myelopathy when aged 18 months or less.

Diagnosis	Number	Percentage
Vertebral malformation	42	18.8
Spinal arachnoid diverticulum	28	12.5
Traumatic fracture of the vertebral column	22	9.8
Atlantoaxial instability	18	8.0
Osseous‐associated cervical spondylomyelopathy	17	7.6
Meningomyelitis of unknown origin	11	4.9
Discospondylitis	10	4.5
Intervertebral disc extrusion	10	4.5
Ischemic myelopathy	8	3.6
Spina bifida	8	3.6
Steroid‐responsive meningitis‐arteritis	8	3.6
Multiple diseases	5	2.2
Traumatic avulsion of the brachial plexus	5	2.2
Acute noncompressive nucleus pulposus extrusion	4	1.8
Neoplasia	4	1.8
Syringohydromyelia	4	1.8
Neosporosis	3	1.3
Cranial cervical traumatic contusion	2	0.9
Idiopathic intramedullary hemorrhage	2	0.9
Unknown bilateral symmetrical encephalomyelopathy	2	0.9
Calcinosis circumscripta	1	0.4
Dermoid sinus (type IV)	1	0.4
Extradural inflammatory mass (pseudotumour)	1	0.4
Foreign body (microchip implantation)	1	0.4
Fracture caused by nutritional secondary hyperparathyroidism	1	0.4
Hemophilia A	1	0.4
Multiple cartilaginous exostoses	1	0.4
Osteomyelitis	1	0.4
Paravertebral abscess	1	0.4
Pyogranulomatous meningomyelitis secondary to sepsis	1	0.4
Vascular malformation	1	0.4

### 3.4 Statistical analysis

Clinical features that remained statistically significant on multivariable logistic regression analyses for each final diagnosis containing 10 or more dogs are detailed below and summarized in Table 3. Results from the preliminary univariable logistic regression analyses are available in Table S1. No variables were correlated based on Pearson's correlation coefficient.

**TABLE 3:** Results of multivariable logistic regression analyses evaluating associations between clinical features and final diagnoses with n ≥ 10.

	Age (months)	BW (kg)	Screw‐tailed and pug breeds	Onset of clinical signs	Duration of clinical signs (days)	Progression of clinical signs	Spinal cord segment	Ambulatory status	Spinal hyperesthesia	Lateralization of clinical signs	Incontinence
VM	Younger *P* = .004 OR = 0.84 (0.74‐0.94)		Screw‐tailed *P* < .001 OR = 4.25 (1.81‐9.98)	Chronic *P* = .005 OR = 3.67 (1.48‐9.08)			T3‐L3 *P* = .001 OR = 7.41 (2.21‐24.77)				
SAD			Screw‐tailed *P* = .008 OR = 3.50 (1.39‐8.78)		Longer *P* < .001 OR = 1.01 (1.00‐1.02)				Nonpainful *P* = .03 OR = 0.18 (0.04‐0.81)		
Fracture				Not included	Not included	Non‐prog. *P* = .001 OR = 0.16 (0.05‐0.49)		Non‐amb. *P* = .02 OR = 0.28 (0.10‐0.85)	Painful *P* < .001 OR = 8.68 (2.47‐30.51)		
AAI		Lower *P* = .002 OR = 0.84 (0.75‐0.94)	Other breeds *P* = .02 OR = 0.08 (0.01‐0.64)				Not included				
OA‐CSM		Higher *P* < .001 OR = 1.08 (1.05‐1.12)									
MUO											
IVDE	Older *P* = .02 OR = 2.68 (1.18‐6.09)		Screw‐tailed *P* = .02 OR = 1006.0 (3.50‐286 818.0)		Shorter *P* = .04 OR = 0.85 (0.73‐0.99)						
Discospondylitis									Painful *P* = .01 OR = 7.72 (1.60‐37.32)		

*Note*: Results displayed are those that retained statistical significance (*P*‐value <.05) following a manual backwards stepwise removal approach. Values are OR (95% CI); *P* values reported to 2 s.f.; OR reported to 2 d.p.; Not included denotes clinical feature not included in the multivariable model, regardless of whether it reached significance in the univariable model.

Abbreviations: AAI, atlantoaxial instability; BW, bodyweight; CI, confidence interval; d.p., decimal places; IVDE, intervertebral disc extrusion; MMUO, meningomyelitis of unknown origin; non‐amb., non‐ambulatory; non‐prog., non‐progressive; OA‐CSM, osseous‐associated cervical spondylomyelopathy; OR, odds ratio; SAD, spinal arachnoid diverticulum; s.f., significant figures; VM, vertebral malformation.

#### 3.4.1 Vertebral malformation

Vertebral malformation was associated with age (*P* = .004, OR = 0.84, 95% CI: 0.74‐0.94), screw‐tailed and pug breeds (*P* < .001, OR = 4.25, 95% CI: 1.81‐9.98), onset of clinical signs (*P* = .005, OR = 3.67, 95% CI: 1.48‐9.08), and the spinal cord segment affected (T3‐L3, *P* = .001, OR = 7.41, 95% CI: 2.21‐24.77). As such, a final diagnosis of vertebral malformation was associated with younger, screw‐tailed, and pug breeds displaying chronic signs of T3‐L3 myelopathy.

#### 3.4.2 Spinal arachnoid diverticulum

Spinal arachnoid diverticulum was associated with duration of clinical signs (*P* < .001, OR = 1.01, 95% CI: 1.00‐1.02), screw‐tailed and pug breeds (*P* = .008, OR = 3.50, 95% CI: 1.39‐8.78), and spinal hyperesthesia (*P* = .03, OR = 0.18, 95% CI: 0.04‐0.81). As such, a final diagnosis of SAD was associated with screw‐tailed and pug breeds displaying a longer duration of nonpainful clinical signs.

#### 3.4.3 Traumatic fracture

Onset and duration of clinical signs were excluded from the multivariable analysis, given that traumatic fractures will inherently be acute in onset and be presented promptly. Traumatic fracture of the vertebral column was associated with progression of clinical signs (deterioration; *P* = .001, OR = 0.16, 95% CI: 0.05‐0.49), ambulatory status (*P* = .02, OR = 0.28, 95% CI: 0.10‐0.85), and spinal hyperesthesia (*P* < .001, OR = 8.68, 95% CI: 2.47‐30.51). As such, a final diagnosis of traumatic fracture was associated with nonambulatory status and painful, nonprogressive clinical signs.

#### 3.4.4 Atlantoaxial instability

Spinal cord segment affected was excluded from the multivariable analysis, because by virtue of the anatomic location, AAI will display signs consistent with a C1‐C5 myelopathy. Atlantoaxial instability was associated with bodyweight (*P* = .002, OR = 0.84, 95% CI: 0.75‐0.94) and screw‐tailed and pug breeds (*P* = .02, OR = 0.08, 95% CI: 0.01‐0.64). As such, a final diagnosis of AAI was associated with a lower bodyweight, whereas screw‐tailed breeds and pugs were protected.

#### 3.4.5 Osseous‐associated cervical spondylomyelopathy

Osseous‐associated cervical spondylomyelopathy was associated with higher bodyweight (*P* < .001, OR = 1.08, 95% CI: 1.05‐1.12).

#### 3.4.6 Meningomyelitis of unknown origin

Meningomyelitis of unknown origin was not associated with any clinical features.

#### 3.4.7 Discospondylitis

Discospondylitis was associated with the presence of hyperesthesia on palpation of the vertebral column (*P* = .01, OR = 7.72, 95% CI: 1.60‐37.32).

#### 3.4.8 Intervertebral disc extrusion

Intervertebral disc extrusion was associated with age (*P* = .02, OR = 2.68, 95% CI: 1.18‐6.09), screw‐tailed and pug breeds (*P* = .02, OR = 1006.0, 95% CI: 3.50‐286 818.0), and duration of clinical signs (*P* = .04, OR = 0.85, 95% CI: 0.73‐0.99). As such, a final diagnosis of IVDE was associated with older, screw‐tailed, and pug breeds with a shorter duration of clinical signs.

## 4 DISCUSSION

Our study describes the prevalence of etiologies associated with signs of myelopathy in dogs aged 18 months or less. Vertebral malformation (18.8%) was the most common condition, followed by spinal arachnoid diverticulum (12.5%), traumatic fracture of the vertebral column (9.8%), atlantoaxial instability (8.0%), and OA‐CSM (7.6%), with 31 diagnoses overall. Of the 11 clinical features that underwent logistic regression analyses, classification as a screw‐tailed or pug breed was the feature most frequently associated with a final diagnosis; vertebral malformation (VM), SAD, and IVDE were all more likely in screw‐tailed and pug breeds, whereas AAI was less likely. Two clinical features were not associated with any final diagnosis, lateralization of clinical signs, and incontinence. Recognition of a constellation of clinical features can help clinicians prioritize which common underlying cause is the most likely,^4^ useful both for assisting with the formulation of an appropriate diagnostic plan and aligning clients' expectations regarding possible treatment options and prognosis.

Although a large percentage of screw‐tailed and pug breeds might be neurologically normal despite vertebral malformation,^9^, ^19^ the high prevalence of malformations in these breeds makes it unsurprising that the same breeds are associated with myelopathies. Vertebral malformations are almost exclusively congenital, and so in severe cases causing myelopathy, it follows that these cases were associated with a younger age. The extent of severe vertebral malformations might be further exacerbated during growth, with vertebral growth broadly considered complete by 9 months of age,^20^ and thus acting as another factor contributing to their presentation at a younger age within this cohort of dogs. It is also important to note that the prevalence of final diagnoses in our study, with VM the most common, likely reflects the popularity of screw‐tailed and pug breeds within the United Kingdom. A recent study surveying the breed composition of the UK canine population identified that almost 35% of dogs aged 12 months or less were categorized as brachycephalic breeds.^21^ As a result, the prevalence of etiologies underlying myelopathies in juvenile dogs might be expected to differ among geographic locations, reflecting that region's breed profile.

The pathogenesis of SAD formation has not been definitively elucidated; however, hypotheses include microinstability arising from vertebral malformation resulting in chronic trauma,^22^, ^23^, ^24^ congenital meningeal malformation,^25^, ^26^ and arachnoiditis.^27^ In the present study, previous spinal disease consistent with arachnoiditis was not reported, leaving microinstability and congenital meningeal malformation the more likely diagnoses. Differentiation between the latter 2 might be aided, in part, by serial imaging, to ascertain if SAD formation is congenital or acquired in this subset of juvenile dogs. Given that a diagnosis of SAD was associated with the screw‐tailed and pug breed group, among which vertebral malformations are common, this might support the microinstability hypothesis. However, SADs also occur in dogs without vertebral malformations,^28^, ^29^ bringing into question the validity of this hypothesis. A diagnosis of SAD was also associated with a longer duration of nonpainful clinical signs, consistent with previous reports regarding the duration of clinical signs.^28^, ^29^, ^30^ Previous studies have reported incontinence (urinary or fecal or both) in approximately 8% of cases,^28^, ^30^ whereas others have reported a much higher prevalence between 47% and 59%.^31^ In our study, incontinence was seen in 16.7% of dogs with a SAD. Incontinence was not associated with a diagnosis of SAD in this cohort of dogs. As such, this suggests that a diagnosis of SAD is no more or less likely when incontinence is reported. Constrictive myelopathy (CM) or meningeal fibrosis did not feature as a final diagnosis in any of the 224 dogs in our study. This fits with previous reports, in which the median age of onset has ranged from 7.5 to 7.7 years,^31^, ^32^ whereas the earliest reported age was 2 years.^32^ The pathogenesis of constrictive myelopathy remains unknown,^31^ although CM and SAD might belong to a spectrum of the same disease process.^31^, ^33^ Sequential development could occur, with CM identified early in the disease course and subsequent widening of the subarachnoid space resulting in SAD development later. Our study does not exclude a possible sequential theory of development; it seems unlikely given that there were no diagnoses of CM in this cohort of juvenile dogs. However, this inference must be taken cautiously because differentiation of CM and SADs based on imaging can be challenging,^31^ particularly when the latter might be multilobulated.^34^ As such, there might have been final diagnoses of SAD included in our study that could be classed as CM.

A final diagnosis of AAI was associated with a lower bodyweight, whereas screw‐tailed breeds and pugs were protected. This is in agreement with previous studies in which AAI is common in low bodyweight toy‐breed dogs.^7^ In addition, the fact that AAI was neither associated with an acute nor chronic presentation is consistent with its inherent heterogeneity with some dogs presenting with sudden‐onset tetraparesis, whereas others display signs of intermittent cervical hyperesthesia.

Intervertebral disc extrusion was the joint seventh most frequent diagnosis in dogs presenting with myelopathic signs when aged 18 months or less. As such, this supports the study hypothesis that IVDE is uncommon in juvenile dogs. The majority of IVDE lesions were within the thoracolumbar region of the vertebral column, with 20% located in the cervical region.

Meningomyelitis of unknown origin was the only final diagnosis not associated with any clinical feature. Many conditions have a typical “fingerprint” of clinical signs; the lack of distinct clinical features could be interpreted in a number of ways. First, MMUO should be included on the list of differential diagnoses for any myelopathy; second, the umbrella‐term diagnosis possibly incorporates a heterogeneous group of conditions resulting in wide‐ranging presentations; or third, perhaps the number of dogs within the diagnosis group might have generated insufficient power to reach statistical significance. The heterogeneity of meningoencephalitis of unknown origin has been widely discussed in the literature,^35^ and so it is not unexpected that this is shared by its spinal cord counterpart. Only 1 published study has described MMUO, in which they also found a spread of presentations, with an almost even split between acute and chronic onset of clinical signs, with their duration ranging from 1 to 90 days.^36^

The study's main limitation was the potential for underreporting of certain clinical features, such as the level of continence, because of the retrospective nature of data retrieval from the medical records; as a result, associations between certain clinical features and a final diagnosis might not have been identified. As is common to most retrospective studies, data regarding the onset and duration of clinical signs are dependent upon recall from either the owner or from the clinical records of the referring veterinarian, both of which are unlikely to be wholly accurate; however, this effect would be seen evenly across all cases in the study. A further limitation is the lack of histopathologic examination to confirm the final diagnoses; this might be most relevant when considering the imaging‐based diagnosis of SAD and that of similar conditions, such as CM. Finally, the inclusion criterion requiring a final diagnosis might bias the data toward more frequently seen and well‐characterized conditions, falsely elevating their prevalence. However, given the number of obscure conditions without a final diagnosis excluded from the study was likely low, their hypothetical inclusion would not be expected to substantially affect the subsequent logistic regression analyses undertaken for final diagnoses with 10 or more cases.

## CONFLICT OF INTEREST DECLARATION

Authors declare no conflict of interest.

## OFF‐LABEL ANTIMICROBIAL DECLARATION

Authors declare no off‐label use of antimicrobials.

## INSTITUTIONAL ANIMAL CARE AND USE COMMITTEE (IACUC) OR OTHER APPROVAL DECLARATION

Approved by the Ethics Committee of the University of Liverpool.

## HUMAN ETHICS APPROVAL DECLARATION

Authors declare human ethics approval was not needed for this study.

## Supporting information


**Table S1.** Results of univariable logistic regression analyses evaluating associations between clinical features and final diagnoses with n ≥ 10. Results highlighted in bold were *P* < .25 and passed into the multivariable model. OR, odds ratio; 95% CI, 95% confidence interval; *P* values reported to 2 s.f.; OR reported to 2 d.p.; ref, reference subcategory, with which subsequent subcategories were compared.
