# Supplementary material for: Prevalence, clinical presentation, and etiology of myelopathies in 224 juvenile dogs
Source: J Vet Intern Med. 2024 Mar 14;38(3):1598–607. doi: 10.1111/jvim.17045 (PMC11099773; doi:10.1111/jvim.17045)
Supplement: Supplementary file 1 — Table S1. Results of univariable logistic regression analyses evaluating associations between clinical features and final diagnoses with n ≥ 10. Results highlighted in bold were P < .25 and passed into the multivariable model. OR, odds ratio; 95% CI, 95% confidence interval; P values reported to 2 s.f.; OR reported to 2 d.p.; ref, reference subcategory, with which subsequent subcategories were compared. [file JVIM-38-1598-s001.docx]

|  | **Vertebral malformation** | | | | **Spinal arachnoid diverticulum** | | | | **Fracture** | | | |
| --- | --- | --- | --- | --- | --- | --- | --- | --- | --- | --- | --- | --- |
|  | p | OR | 95% CI | | p | OR | 95% CI | | p | OR | 95% CI | |
|  |  |  | Lower | Upper |  |  | Lower | Upper |  |  | Lower | Upper |
| Age | **0.006** | 0.87 | 0.79 | 0.96 | 0.86 | 1.01 | 0.92 | 1.11 | **0.19** | 1.07 | 0.97 | 1.18 |
| Bodyweight | **0.13** | 0.97 | 0.94 | 1.01 | **0.058** | 0.95 | 0.91 | 1.00 | 0.83 | 1.00 | 0.97 | 1.04 |
| Screw-tail breed | **<.001** | 7.04 | 3.30 | 15.06 | **0.002** | 3.63 | 1.59 | 8.31 | **0.01** | 0.07 | 0.009 | 0.53 |
| Onset | **0.003** | 3.2 | 1.49 | 6.89 | **0.002** | 5.88 | 1.97 | 17.57 | **<.001** | 0.07 | 0.015 | 0.29 |
| Duration of clinical signs | 0.98 | 1 | 0.99 | 1.01 | **<.001** | 1.01 | 1.01 | 1.02 | **0.013** | 0.95 | 0.91 | 0.99 |
| Progression |  |  |  |  |  |  |  |  |  |  |  |  |
| Static | **0.25** | *ref* | ~ | ~ | 0.73 | *ref* | ~ | ~ | **0.003** | *ref* | ~ | ~ |
| Deteriorating | 0.059 | 2.33 | 0.97 | 5.64 | 0.27 | 0.63 | 0.27 | 1.44 | <.001 | 0.15 | 0.054 | 0.40 |
| Improving | 0.93 | 0.91 | 0.10 | 8.25 | 0.57 | 0.54 | 0.061 | 4.67 | 0.36 | 0.36 | 0.042 | 3.10 |
| Waxing-waning | 0.35 | 2.33 | 0.40 | 13.48 | 0.99 | 0 | 0 | . | 0.99 | 0 | 0 | . |
| Spinal cord segment |  |  |  |  |  |  |  |  |  |  |  |  |
| C1-C5 | **0.001** | *ref* | ~ | ~ | 0.45 | *ref* | ~ | ~ | 0.36 | *ref* | ~ | ~ |
| C6-T2 | 0.99 | 0 | 0 | . | 0.99 | 0 | 0 | . | 0.68 | 0.63 | 0.066 | 5.91 |
| T3-L3 | <.001 | 7.08 | 2.37 | 21.13 | 0.17 | 0.56 | 0.24 | 1.28 | 0.25 | 1.99 | 0.62 | 6.40 |
| L4-S3 | 0.55 | 1.72 | 0.29 | 10.26 | 0.99 | 0 | 0 | . | 0.075 | 3.93 | 0.87 | 17.70 |
| Multifocal | 0.74 | 0.69 | 0.07 | 6.53 | 0.11 | 0.18 | 0.022 | 1.45 | 0.99 | 0 | 0 | . |
| Ambulatory status | **0.003** | 9.27 | 2.16 | 39.69 | **0.018** | 11.52 | 1.53 | 86.76 | **<.001** | 0.16 | 0.06 | 0.42 |
| Spinal hyperesthesia | **0.007** | 0.30 | 0.13 | 0.72 | **0.004** | 0.11 | 0.026 | 0.49 | **<.001** | 7.34 | 2.59 | 20.78 |
| Lateralisation of clinical signs | 0.30 | 0.61 | 0.24 | 1.54 | **0.084** | 0.27 | 0.062 | 1.19 | **0.22** | 0.39 | 0.087 | 1.74 |
| Incontinence | 0.94 | 0.96 | 0.31 | 3.00 | 0.40 | 1.65 | 0.51 | 5.28 | 0.90 | 0.91 | 0.20 | 4.18 |

**Supplementary Table 1 – Results of univariable logistic regression analyses evaluating associations between clinical features and final diagnoses with n ≥10.** Results highlighted in bold were p<0.25 and passed into the multivariable model. p – p-value; OR – odds ratio; 95% CI – 95% confidence interval; P values reported to 2 s.f.; OR reported to 2 d.p.; ref – reference sub-category, with which subsequent sub-categories were compared.

|  | **Atlantoaxial instability** | | | | **OA-CSM** | | | | **MMUO** | | | |
| --- | --- | --- | --- | --- | --- | --- | --- | --- | --- | --- | --- | --- |
|  | p | OR | 95% CI | | p | OR | 95% CI | | p | OR | 95% CI | |
|  |  |  | Lower | Upper |  |  | Lower | Upper |  |  | Lower | Upper |
| Age | **0.18** | 0.92 | 0.81 | 1.04 | 0.6 | 1.03 | 0.92 | 1.15 | 0.49 | 1.05 | 0.92 | 1.20 |
| Bodyweight | **<.001** | 0.80 | 0.71 | 0.91 | **<.001** | 1.08 | 1.05 | 1.12 | 0.66 | 0.99 | 0.94 | 1.04 |
| Screw-tail breed | **0.02** | 0.09 | 0.01 | 0.68 | 0.99 | 0.00 | 0.00 | . | 0.56 | 1.44 | 0.43 | 4.88 |
| Onset | 0.36 | 0.63 | 0.24 | 1.67 | **0.18** | 2.08 | 0.71 | 6.10 | **0.025** | 0.17 | 0.04 | 0.80 |
| Duration of clinical signs | 0.28 | 1.00 | 1.00 | 1.01 | 0.81 | 1.00 | 0.99 | 1.01 | **0.085** | 0.95 | 0.90 | 1.01 |
| Progression |  |  |  |  |  |  |  |  |  |  |  |  |
| Static | 0.48 | *ref* | ~ | ~ | 0.82 | *ref* | ~ | ~ | 0.82 | *ref* | ~ | ~ |
| Deteriorating | 0.67 | 1.29 | 0.39 | 4.22 | 0.34 | 2.15 | 0.45 | 10.23 | 0.34 | 2.15 | 0.45 | 10.23 |
| Improving | 0.66 | 2.67 | 0.17 | 16.63 | 0.99 | 0.00 | 0.00 | . | 0.99 | 0.00 | 0.00 | . |
| Waxing-waning | **0.13** | 4.29 | 0.66 | 27.79 | 0.99 | 0.00 | 0.00 | . | 0.99 | 0.00 | 0.00 | . |
| Spinal cord segment |  |  |  |  |  |  |  |  |  |  |  |  |
| C1-C5 | **0.005** | *ref* | ~ | ~ | **0.022** | *ref* | ~ | ~ | 0.44 | *ref* | ~ | ~ |
| C6-T2 | 0.99 | 0 | 0 | . | 0.12 | 2.62 | 0.77 | 8.88 | 0.99 | 0.00 | 0.00 | . |
| T3-L3 | <0.001 | 0.03 | 0 | 0.21 | 0.019 | 0.15 | 0.03 | 0.73 | **0.18** | 4.23 | 0.51 | 35.25 |
| L4-S3 | 0.99 | 0 | 0 | . | 0.99 | 0.00 | 0.00 | . | 0.99 | 0.00 | 0.00 | . |
| Multifocal | 0.06 | 0.13 | 0.02 | 1.09 | 0.77 | 0.78 | 0.15 | 4.10 | **0.056** | 9.67 | 0.95 | 98.77 |
| Ambulatory status | 0.90 | 1.07 | 0.37 | 3.13 | **0.059** | 7.16 | 0.93 | 55.16 | 0.96 | 0.96 | 0.25 | 3.76 |
| Spinal hyperesthesia | 0.34 | 1.63 | 0.60 | 4.41 | **0.11** | 0.35 | 0.10 | 1.27 | 0.53 | 0.65 | 0.17 | 2.51 |
| Lateralisation of clinical signs | **0.046** | 2.80 | 1.02 | 7.69 | 0.78 | 0.84 | 0.23 | 3.05 | **0.18** | 2.38 | 0.67 | 8.53 |
| Incontinence | 0.99 | 0 | 0 | . | 0.58 | 0.55 | 0.07 | 4.39 | 0.93 | 0.91 | 0.11 | 7.50 |

**Supplementary Table 1 *continued* – Results of univariable logistic regression analyses evaluating associations between clinical features and final diagnoses with n ≥10.** Results highlighted in bold were p<0.25 and passed into the multivariable model. p – p-value; OR – odds ratio; 95% CI – 95% confidence interval; P values reported to 2 s.f.; OR reported to 2 d.p.; ref – reference sub-category, with which subsequent sub-categories were compared.

|  | **Intervertebral disc extrusion** | | | | **Discospondylitis** | | | |
| --- | --- | --- | --- | --- | --- | --- | --- | --- |
|  | p | OR | 95% CI | | p | OR | 95% CI | |
|  |  |  | Lower | Upper |  |  | Lower | Upper |
| Age | **<.001** | 1.58 | 1.27 | 1.96 | 0.35 | 1.07 | 0.93 | 1.23 |
| Bodyweight | 0.53 | 0.98 | 0.92 | 1.04 | 0.75 | 1.01 | 0.97 | 1.05 |
| Screw-tail breed | **0.013** | 7.41 | 1.54 | 35.80 | 0.39 | 1.74 | 0.49 | 6.21 |
| Onset | 0.99 | 0.00 | 0.00 | . | 0.74 | 1.24 | 0.34 | 4.53 |
| Duration of clinical signs | **0.06** | 0.88 | 0.77 | 1.01 | 0.76 | 1.00 | 0.99 | 1.01 |
| Progression |  |  |  |  |  |  |  |  |
| Static | 0.77 | *ref* | ~ | ~ | 0.53 | *ref* | ~ | ~ |
| Deteriorating | 0.54 | 1.64 | 0.33 | 8.14 | 0.21 | 3.85 | 0.47 | 31.43 |
| Improving | 0.99 | 0.00 | 0.00 | . | 0.99 | 0.00 | 0.00 | . |
| Waxing-waning | 0.29 | 3.88 | 0.32 | 47.72 | 0.16 | 7.88 | 0.45 | 138.58 |
| Spinal cord segment |  |  |  |  |  |  |  |  |
| C1-C5 | 0.88 | *ref* | ~ | ~ | 0.62 | *ref* | ~ | ~ |
| C6-T2 | 0.99 | 0.00 | 0.00 | . | 1 | 1.00 | 0.00 | . |
| T3-L3 | 0.28 | 2.40 | 0.49 | 11.70 | 0.99 | . | 0.00 | . |
| L4-S3 | 0.99 | 0.00 | 0.00 | . | 0.99 | . | 0.00 | . |
| Multifocal | 0.99 | 0.00 | 0.00 | . | 0.99 | . | 0.00 | . |
| Ambulatory status | **0.1** | 0.34 | 0.10 | 1.23 | 0.80 | 0.84 | 0.21 | 3.35 |
| Spinal hyperesthesia | **0.035** | 4.41 | 1.11 | 17.57 | 0.011 | 7.72 | 1.60 | 37.32 |
| Lateralisation of clinical signs | 0.43 | 0.43 | 0.05 | 3.46 | 0.99 | 0.00 | 0.00 | . |
| Incontinence | 0.99 | 0.00 | 0.00 | . | 0.99 | 0.00 | 0.00 | . |

**Supplementary Table 1 *continued* – Results of univariable logistic regression analyses evaluating associations between clinical features and final diagnoses with n ≥10.** Results highlighted in bold were p<0.25 and passed into the multivariable model. p – p-value; OR – odds ratio; 95% CI – 95% confidence interval; P values reported to 2 s.f.; OR reported to 2 d.p.; ref – reference sub-category, with which subsequent sub-categories were compared.
